# Genome-wide identification and expression analysis of *EPF/EPFL* gene family in *Populus trichocarpa*

**DOI:** 10.3389/fgene.2024.1432376

**Published:** 2024-07-18

**Authors:** Sha Liu, Ting Chen, Xin Li, Junjun Cui, Yinshuai Tian

**Affiliations:** School of Landscape and Ecological Engineering, Hebei University of Engineering, Handan, China

**Keywords:** *EPF/EPFL* gene family, poplar, gene expression, drought, stomata

## Abstract

The Epidermal Patterning Factor/EPF-like (EPF/EPFL) family encodes a specific type of secreted protein in plants and plays an important role in plant growth and development, especially in the process of morphogenesis. To investigate the characteristics of *EPF/EPFL* gene family members and their regulatory functions in stomatal development of *Populus trichocarpa*, a total of 15 *EPF/EPFL* family genes were identified. Then the gene structure, chromosome location, phylogenetic relationship, protein conserved domain and gene expression profile were analyzed. According to phylogenetic analysis, PtEPF/EPFL can be classified into four groups. The gene structure and protein conservation motifs within the EPF family indicate the high conservation of the *PtEPF/EPFL* sequence. The promoter region of *PtEPF/EPFL* was found to contain cis-elements in response to stress and plant hormones. In addition, RT-qPCR results indicated that the *PtEPF/EPFL* have a differentially expressed in different tissues. Under drought stress treatment, a substantial upregulation was observed in the majority of *PtEPF/EPFL* members, suggesting their potential involvement in drought response. These results provide a theoretical basis for future exploration of the characteristics and functions of more *PtEPF/EPFL* genes.

## 1 Introduction

Drought stress is the most important environmental constraint affecting plant growth and distribution (Zhu, 2002; Kissoudis et al., 2016). The majority of water absorbed by plants is lost to the air via stomata and reducing the transpiration water loss through stomata is one of the effective strategy to improve plant drought resistance (Lawson and Blatt, 2014). Stomata play a crucial role in regulating both carbon dioxide intake and water loss in plants, which determines the productivity and water use efficiency (WUE) of plants (Wang et al., 2016; Buckley et al., 2019). By changing their stomata, plants could optimize the absorption of carbon dioxide by photosynthesis while minimizing water loss, thus drought tolerance and WUE of plants were improved (Bertolino et al., 2019; Zhao et al., 2020).

The development of stomata is a complex process, which involving the regulation of signal transduction, cell fate transformation and asymmetric division. Researches have revealed that the EPIDERMAL PATTERNING FACTOR (EPF) family consists of a group of cysteine-rich secreted peptides and plays a prominent role in stomatal development (Kondo et al., 2010; Katsir et al., 2011). The *EPF* family in *Arabidopsis* consists of 11 members, including *EPF1*, *EPF2*, and *EPFL1-9*. Mature EPF peptides typically exhibit conserved six to eight cysteine residues at the C-terminal, which form intramolecular disulfide bonds that are important for maintaining their spatial structure (Ohki et al., 2011). Some members of the *EPF* family are involved in regulating stomatal density and patterning within the plant epidermis. *AtEPF1* and *AtEPF2* have been identified as negative regulators of stomatal development. Although the amino acid sequences of *AtEPF1* and *AtEPF2* are highly similar, they show different functions in stomatal development. *AtEPF2* regulates the differentiation of pro-epidermal cells into meristemoid mother cell (MMC), while *AtEPF1* regulates the direction of spacing division and produces satellite meristem (Hara et al., 2007; Hara et al., 2009; Hunt and Gray, 2009). *AtEPFL9*, also known as *Stomagen,* functions as a positive regulator of leaf stomatal density while exerting an antagonistic effect on *AtEPF1* and *AtEPF2* (Hunt et al., 2010; Sugano et al., 2010). As an important member of the *EPF* family, the *CHAL* subfamily has similar functions to *EPF1* and *EPF2* and is a negative regulator of stomatal development (Abrash and Bergmann, 2010). In addition, *EPF* genes are also involved in the regulation of plant inflorescence structure, leaf morphology, pollen tube elongation and awn development (Abrash et al., 2011; Uchida et al., 2012; Xiong et al., 2022).

Genetic and physiological analysis demonstrated that *EPF* genes have great potential in improving plant WUE and drought tolerance by regulating stomata development. It was found that overexpression *EPF1* reduced stomatal density and improved WUE and drought tolerance of plants in barley, rice, and wheat (Hughes et al., 2017; Caine et al., 2019; Dunn et al., 2019). Overexpression of *StEPF2* and *PdEPF2* in *Arabidopsis* leads to a significant reduction in stomatal density. However, under drought stress conditions, these plants maintain higher photosynthetic rates, photosystem II efficiency, and instantaneous WUE compared to wild-type (WT) plants (Wang et al., 2016; Wang et al., 2020). Similar results were also obtained in *Arabidopsis* (Yu et al., 2008; Franks et al., 2015), maize (Xiang et al., 2021), tomato (Li et al., 2019; Li et al., 2020) and poplar (Wang et al., 2016; Li et al., 2021). Recent studies have revealed the crucial functions performed by members of the *EPF* gene family in the growth and development of plants. Overexpression of *TaEPFL1* can shorten the filament of transgenic *Arabidopsis thaliana* and cause abnormal development of horn fruit, thus affecting the development of male stamens (Sun et al., 2019). *EPFL2* and *EPFL9* contribute to the coordination of ovule patterning, influencing seed number alongside gynoecium and fruit growth via the *ERECTA* family receptors (Kawamoto et al., 2020). These studies suggest that genes regulating stomatal development are potential candidate genes for improving plants WUE by reducing stomatal density.

Poplar is one of the most important plantation species in temperate regions of the world. It has the advantages of fast growth rate, strong adaptability, early timber formation and large wood accumulation, etc. With the completion of whole genome sequencing of *Populus trichocarpa* and *Populus euphratica* (Ma et al., 2013; Zhang et al., 2014), poplar has become an ideal woody model plant. *EPF* gene family is an important factor in the regulation of stomatal development. In this study, the identification of the poplar *EPF*/*EPFL* gene family, covering aspects such as physicochemical property analysis, chromosomal location, phylogenetic analysis, gene structure, conserved domains, and cis-regulatory elements in the promoter. Furthermore, the study explored the tissue-specific expression patterns and transcriptional responses to drought stress and abscisic acid (ABA) of this gene family. Identification and analysis of *EPF*/*EPFL* family gene phenotype in poplar are helpful to explore its role in stomatal development, which provided a theoretical basis for breeding poplar varieties with high WUE and fast growth rate.

## 2 Materials and methods

### 2.1 Genome data used in this study

*P. trichocarpa* genome and annotation information were downloaded from the Ensembl Plants database (https://plants.ensembl.org). The genome data for the 11 EPF family in *A. thaliana* were downloaded from TAIR (https://www.arabidopsis.org). Download genome data from the Phytozome (v4.1) database (https://phytozome-next.jgi.doe.gov/), which includes genomes of *Physcomitrella patens*, *Selaginella moellendorffii*, *Sorghum bicolor*, *Medicago truncatula*, *Carica papaya*, *Picea abies*, and *Oryza sativa*.

### 2.2 *EPF* gene family identification and chromosomal mapping

The EPF Hidden Markov Model (HMM) profile of the EPF domain (pfam17181) was used to blast against the *P. trichocarpa* reference genome to identify EPF genes. A total of 15 putative EPF-encoding genes were identified. Physicochemical properties such as CDS and protein lengths, molecular weights (MW), isoelectric points (IP), and hydrophilicity analysis were forecasted using ExPasy ProtParam (https://web.expasy.org/protparam/). SingnalP-4.1 was used to predict the signal peptide of PtEPF/EPFL protein. Using the online website WoLFPOSRT (https://wolfpsort.hgc.jp/) for subcellular localization prediction. According to the gff3 format annotation file downloaded from the poplar genome database, the size of each member chromosome and the gene location information of each member were obtained. Then the chromosomal localization and syntenic analysis were performed using TBtools software (Chen et al., 2020).

### 2.3 Phylogenetic tree, gene structure and conserved motifs analysis

To identify EPF genes across various species, TBLASTN searches were conducted against genomic databases using amino acid sequences encoded by AtEPF/EPFL genes as references. Sequence comparisons were performed using MEGA7, and a phylogenetic tree was constructed using the Neighbor-Joining (NJ) method with 1,000 repetitions. The gene structure was mapped in TBTools based on the downloaded genome annotation information. The protein conserved motifs of PtEPF/EPFL gene family were predicted using the MEME online program (https://meme-suite.org), with default algorithm parameters (Bailey et al., 2015). The maximum number of motifs was set to 10 and visualized using TBtools software (Chen et al., 2020)

### 2.4 Analysis of cis-acting elements in *PtEPF/EPFL* promoters

Based on the identification of the obtained gene IDs, the promoter sequences (2,000 bp upstream of the start codon) of *PtEPF/EPFL* were extracted by TBtools software. The promoter cis-acting elements of *PtEPF/EPFL* genes were predicted by PlantCARE database (http://bioinformatics.psb.ugent.be/webtools/plantcare/html/) to analyze the cis-elements species, number and function.

### 2.5 Plant materials and sample collections

Uniformly growing one-year-old *P. trichocarpa* trees were selected for this study. Plants grown in the nursery at Beijing Forestry University, Beijing, China (40°000N, 116°200E; 49 m above sea level), in loamy sandy soil (pH = 6.0). Three seedlings with good growth were selected for biological replicates in each pot. For drought treatment, water was withheld from plants for 0, 3, 6, 9, or 12 days. The control group was watered once every 3 days. And young leaves (the first one to three leaves from the shoot apex) were harvested. ABA treatment involved spraying the leaves once with a 250 µM ABA solution, followed by the collection of young leaves at 0, 3, 6, 9, or 12 h post-treatment. Various tissues of one-year-old *Populus L.*, including young leaves, mature leaves, senescent leaves, stems, and roots, were collected. All collected samples were promptly frozen in liquid nitrogen and stored at −80°C for subsequent RNA isolation.

### 2.6 RNA extraction, reverse transcription, and qRT-PCR

Total RNA was extracted using CTAB method, and reverse transcription was carried out according to the instructions of the FastKing RT kit (Tiangen, Beijing, China). The cDNA was used as a template for quantitative real-time polymerase chain reaction (qRT-PCR). Quantitative (R) RT-PCRs were conducted in 96-well plates, each containing 100 ng of template (1 µL), 0.6 µL of forward primer (10 µM), 0.6 µL of reverse primer (10 µM), 10 µL of SYBR Green Master Mix (Tiangen, Beijing, China), 2 µL of ROXTM Reference Dye, and 5.8 µL of RNase-free ddH2O, resulting in a total reaction volume of 20 µL. The amplification was carried out for 40 cycles, with denaturation at 95°C for 10 s, annealing at 55°C for 30 s, and extension at 72°C for 30 s. The reactions were performed in biological triplicates using RNA samples extracted from three independent plant materials, and all experiments were repeated three times. *PtUBQ* was used as the internal reference gene. Transcript levels of all candidate genes were determined using the 2^-△△^CT method, and heatmap visualization was conducted using TBtools (Chen et al., 2020). Primers were designed using Primer6 software, as listed in Supplementary Table S1.

### 2.7 Statistical analyses

All statistical analyses and plots were conducted using SPSS (IBM Corporation, Armonk, NY, United States). Statistical comparisons were performed using one-way analysis of variance (ANOVA) in SPSS. Different letters denote significant differences at a significance level of α = 0.05 (one-way ANOVA). Data were presented as mean ± standard error (SE).

## 3 Results

### 3.1 Identification and physicochemical analysis of the poplar EPF family genes

The gene family was discovered through a search using information probes comprising 11 members of the *EPF/EPFL* family in *Arabidopsis*, along with the conserved domain of the *EPF* family (pfam17181), resulting in the identification of 15 *EPF/EPFL* members in poplar. Detailed information on the gene ID, CDs size, amino acid length, isoelectric point (PI), molecular weight, signal peptide, prediction of subcellular location, and other characteristics of each *PtEPF/EPFL* family member is provided. As shown in Table 1, all EPF/EPFL family proteins are relatively short and are classified as secretory polypeptides, with lengths ranging from 107 aa to 177 aa. Their molecular weights vary from 11.98 to 19.80 kDa, and their isoelectric points range from 4.65 to 10.06. The nomenclature of the *PtEPF/EPFL* gene family was based on the sequences of 11 members of the *EPF/EPFL* family genes in *Arabidopsis*.

**TABLE 1:** The identification and physicochemical analysis of *PtEPF/EPFL* family.

Name	Gene ID	CDs size (nts)	Length(aa)	PI	MW(KDa)	Atomic composition (S)	Signal peptide (Yes/No)	Prediction of subcellular location	Homologs in *Arabidopsis*
PtEPF1-1	Potri.019G102800	360	119	9.11	13.13	C_570_H_907_N_173_O_15813_	Yes	Choloroplast	At2g20875/EPF1
PtEPF1-2	Potri.013G136100	387	128	9.32	14.21	C_617_H_997_N_185_O_17015_	Yes	Choloroplast	At2g20875/EPF1
PtEPF2	Potri.013G116600	351	116	8.63	12.84	C_566_H_883_N_151_O_16015_	Yes	Choloroplast	At1g34245/EPF2
PtEPFL1-1	Potri.007G095400	414	137	8.64	15.40	C_692_H_1038_N_188_O_19111_	No	Extracell	At5g10310/EPFL1
PtEPFL1-2	Potri.005G073700	390	129	8.45	14.26	C_634_H_972_N_174_O_18210_	No	Choloroplast	At5g10310/EPFL1
PtEPFL2	Potri.002G112900	465	154	9.71	17.08	C_745_H_1223_N_221_O_21810_	No	Extracell	At4g37810/EPFL2
PtEPFL3-1	Potri.003G042300	432	143	7.58	14.83	C_646_H_1023_N_179_O_20110_	Yes	Choloroplast	At3g13898/EPFL3
PtEPFL3-2	Potri.003G043200	534	177	4.65	19.80	C_886_H_1407_N_227_O_2736_	No	Choloroplast	At3g13898/EPFL3
PtEPFL4	Potri.013G155500	420	139	9.43	15.64	C_710_H_1094_N_182_O_19013_	No	Extracell	At4g14723/EPFL4
PtEPFL5-1	Potri.008G157300	351	116	9.91	12.61	C_561_H_890_N_162_O_1519_	Yes	Nucleus	At3g22820/EPFL5
PtEPFL5-2	Potri.010G082200	354	117	9.38	12.77	C_566_H_884_N_162_O_1589_	Yes	Extracell	At3g22820/EPFL5
PtEPFL6	Potri.019G128200	420	139	9.02	15.93	C_719_H_1082_N_186_O_19714_	No	Mitochondrion	At2g30370/EPFL6
PtEPFL7	Potri.011G123000	432	143	10.06	15.88	C_698_H_1146_N_204_O_19014_	No	Extracell	At1g71866/EPFL7
PtEPFL8	Potri.018G130700	330	109	9.41	11.98	C_528_H_834_N_144_O_15211_	Yes	Choloroplast	At1g80133/EPFL8
PtEPFL9	Potri.002G249901	324	107	7.59	12.06	C_522_H_821_N_149_O_16010_	Yes	Extracell	At4g12970/EPFL9

### 3.2 Chromosomal localization and syntenic analysis of the *PtEPF/EPFL* genes

**FIGURE 1:**
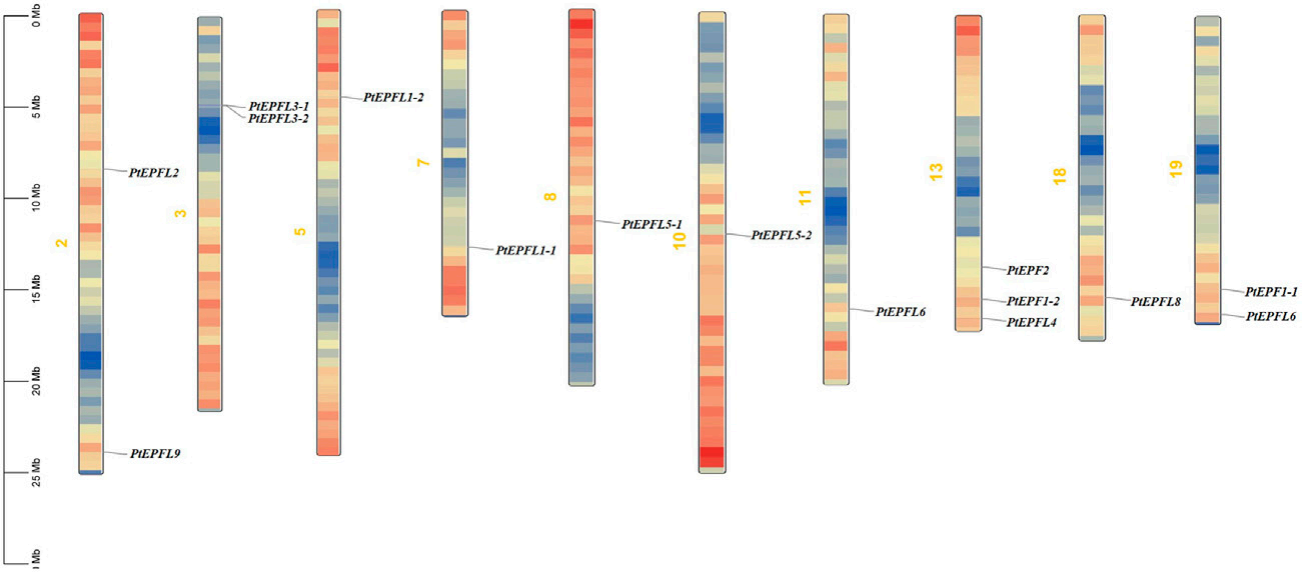
Chromosomal localization of PtEPF/EPFL family in poplar. Size of each chromosome and gene position can be estimated according to the scale on the left of the figure. Chromosome colors represent gene abundance.

**FIGURE 2:**
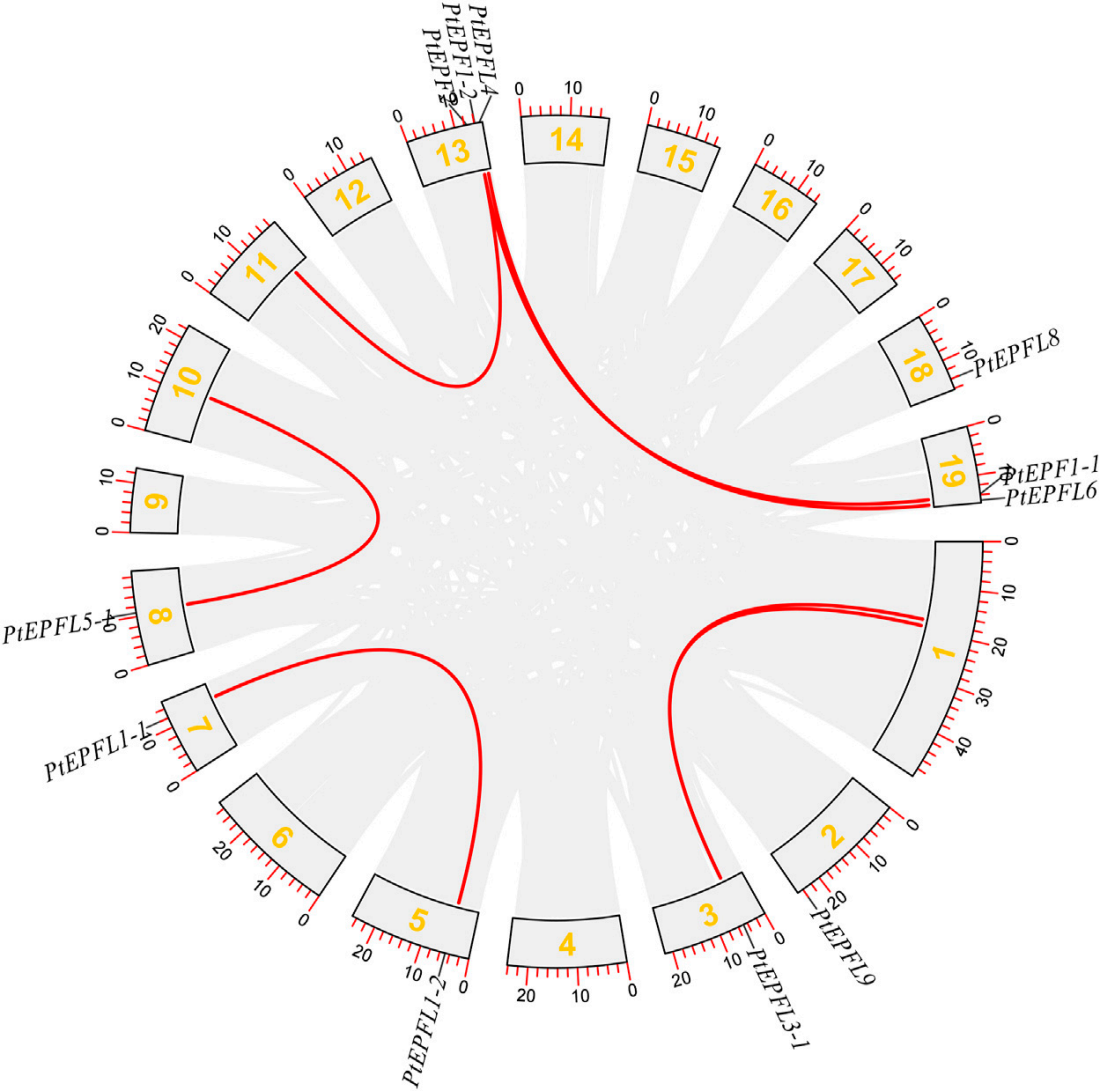
Syntenic analysis of *PtEPF/EPFL* genes. Gray lines indicate all collinearity blocks in the poplar genome, red lines indicates segmental duplicated *PtEPF/EPFL* gene pairs.

The chromosomal localization analysis revealed that the 15 *PtEPF/EPFL* genes were dispersed across ten chromosomes of *P. trichocarpa*, as depicted in Figure 1. The distribution of *PtEPF/EPFL* genes across chromosomes varied, with an uneven range from one to three genes per chromosome. Chromosome three exhibited the highest distribution with three genes, followed by chromosomes 2, 3, and 9, each containing two genes. Additionally, chromosomes 5, 7, 8, 10, and 18 harbored one *PtEPF/EPFL* gene each. To characterize the expansion patterns and replication relationships of the *PtEPF/EPFL* genes, a syntenic analysis of the family genes was performed using the TBtools software. A total of seven colinear gene pairs were identified, including two pairs on chromosome 1/3, one pair on 5/7, one pair on 8/10, one pair on 11/13, and two pairs on 13/19 (Figure 2).

### 3.3 Evolutionary tree analysis of EPF family genes in poplar and other species

**FIGURE 3:**
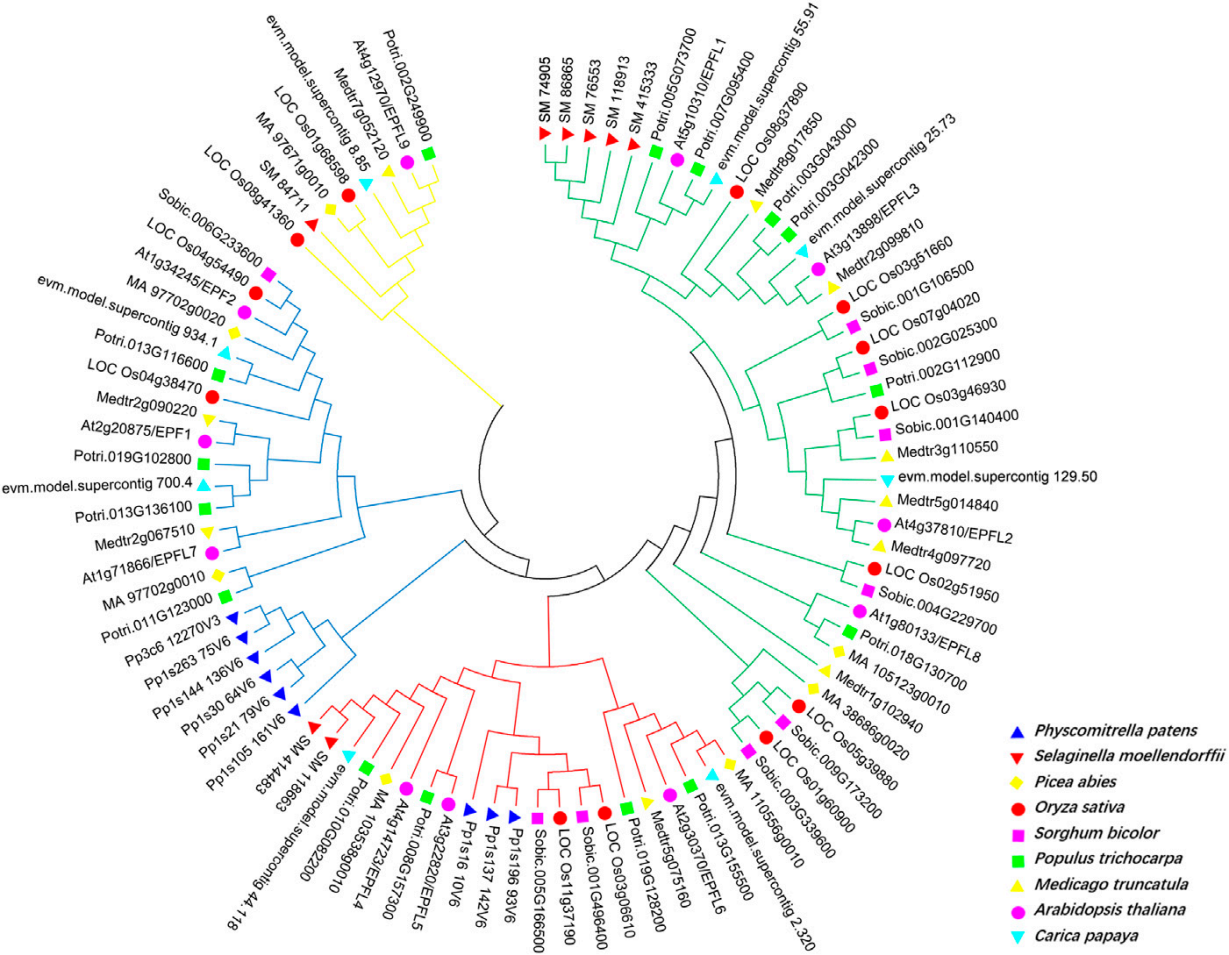
Phylogenetic analysis of EPF family genes in Physcomitrella patens, Selaginella moellendorffii, Picea abies, Oryza sativa, Sorghum bicolor, Populus trichocarpa, Medicago truncatula, *A. thaliana* and Carica papaya. Different species are indicated by different colors.

To investigate the evolutionary relationship of EPF family genes, we selected various representative plant EPF family genes to construct an evolutionary tree. There are 11 AtEPF family members in *Arabidopsis*, and 9, 8, 7, 13, 10, 8, 10, and 15 EPF family members in *P. patens*, *S. moellendorffii*, *P. abies*, *O. sativa*, *S. bicolor*, *C. papaya*, and *P. trichocarpa*, respectively. Using MEGA7 and the Nei *M. truncatula* ghbor-Joining (NJ) method, we conducted multiple sequence alignment of full-length EPF proteins from nine species, and a phylogenetic tree was constructed. The evolutionary tree showed that the EPF gene family can be divided into four groups: EPFL9/Stomagen branch (yellow line), EPF1-EPF2-EPFL7 branch (blue line), EPFL4-6 branch (red line), and EPFL1-3-EPFL8 branch (green line) (Figure 3). Among them, *P. patens* exists only in the branches of EPF1-EPF2-EPFL7 and EPFL4-6, while there were no members of the branches of EPFL9/Stomagen and EPFL1-3-EPFL8, suggesting that these two branches may have originated after pteridophytes.

### 3.4 Analysis of conserved sites of EPF family members

**FIGURE 4:**
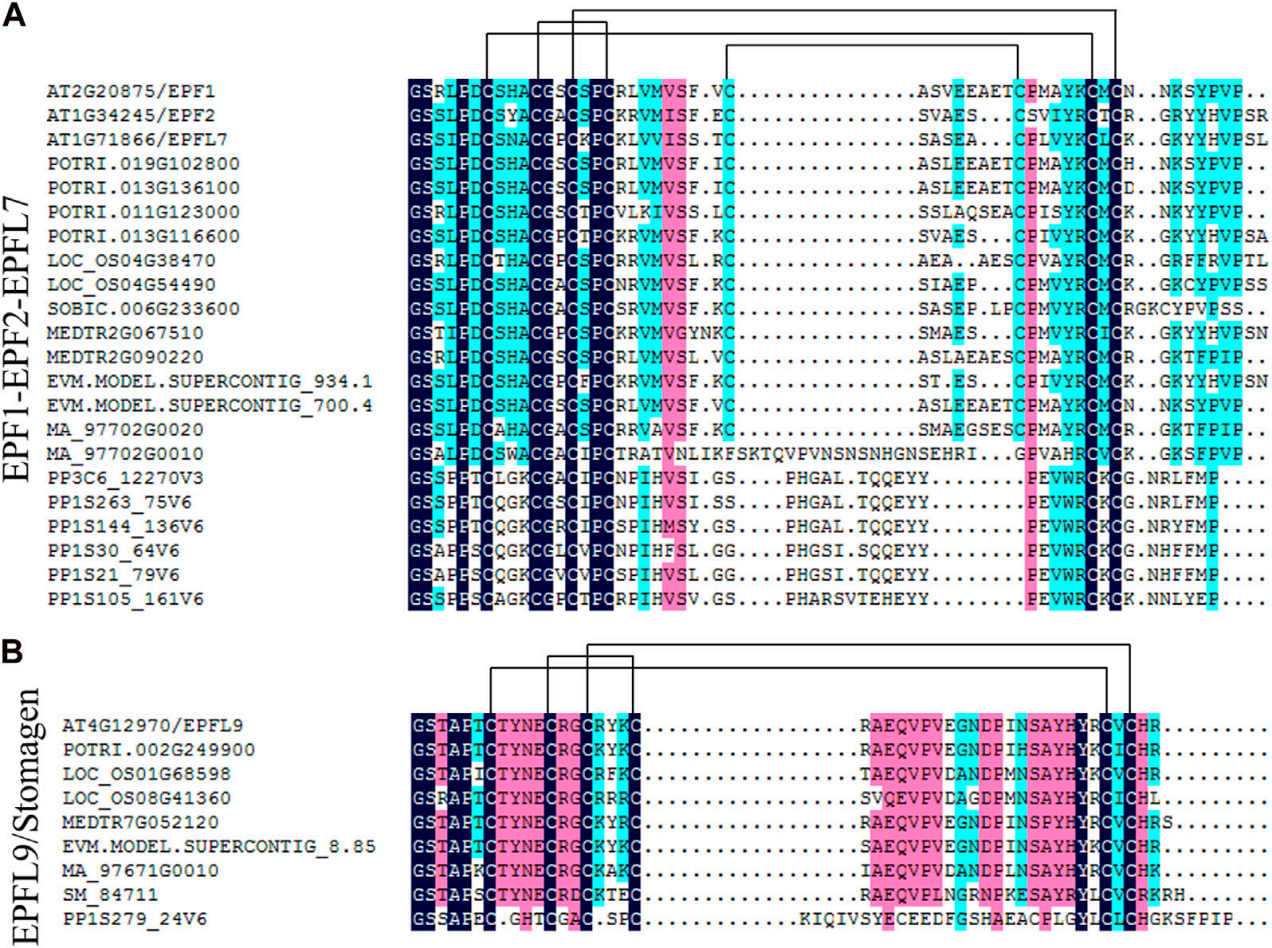
Analysis of conserved of EPF1-EPF2-EPFL7 clade **(A)** and EPFL9/Stomagen clade **(B)** in land plants. Sequence alignment was generated by the DNAMAN. Pairs of cysteine residues forming disulfide bonds predicted for EPF/EPFL family genes are indicated by lines.

Phylogenetic tree analysis showed that EPF1-EPF2-EPFL7 was closely related to EPFL9/Stomagen, and amino acid sequences of the primary protein structure of the two branches were compared. As shown in Figure 4, both the EPF1-EPF2-EPFL7 branch and the EPFL9/Stomagen branch have six conserved cysteine residues in the C-terminal region of the mature peptide, and in the EPF1-EPF2-EPFL7 branch, in addition to the *P. patens*, other species also have two additional conserved cysteine residues.

### 3.5 Gene structure and protein conserved motifs analysis of the *EPF/EPFL* family in poplar

**FIGURE 5:**
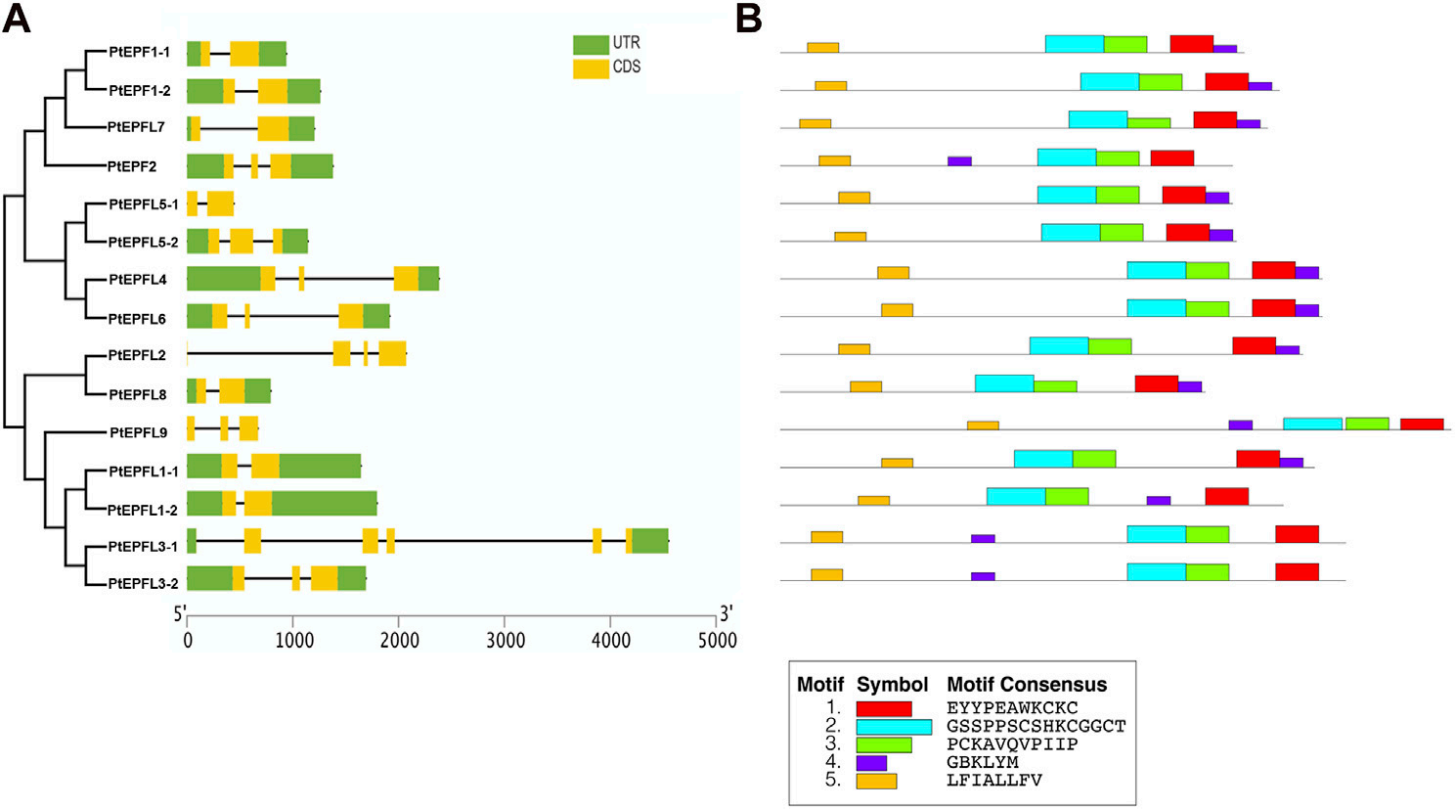
Gene structure and conserved protein motifs analysis of *PtEPF/EPFL.* **(A)** Exon–intron structures of *PtEPF/EPFL*. **(B)** Conserved motifs of PtEPF/EPFL.

Gene structure analysis revealed that the *PtEPF/EPFL* gene family exhibits a range of exon numbers, from 2 to 5. And the *PtEPFL3-1* had the highest number of exons. Moreover, the lengths of the 5′-UTR or 3′-UTR varied among different *PtEPF/EPFL* members (Figure 5A). The results of conserved motif analysis (Figure 5B) showed that PtEPF/EPFL proteins all contained five motif structures, some motif structures were relatively conserved in family genes, and some motifs showed varying degrees of specificity among family members. The PtEPFL9, which correspond to the only positive regulator of stomatal development in *Arabidopsis*, AtEPFL9, exhibited the lowest conservation compared to other PtEPF/EPFL family members. The difference of protein conserved motifs may be the cause of functional diversity of poplar EPF/EPFL family.

### 3.6 Analysis of the *PtEPF/EPFL* gene family promoter

**FIGURE 6:**
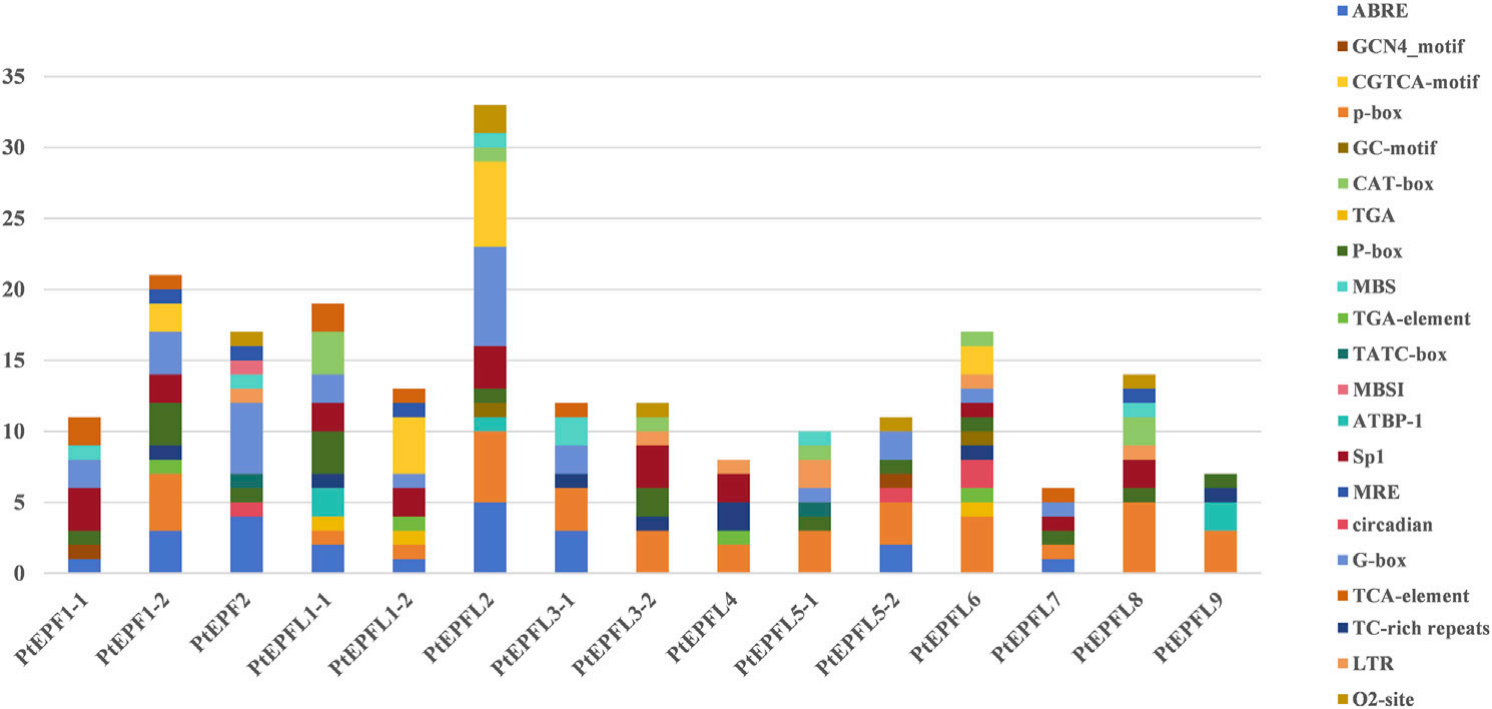
Cis-acting elements were predicted within the 2,000 bp promoter regions of *PtEPF/EPFL*, with different cis-acting elements indicated by differently coloured boxes. Names of cis-acting elements are shown on the right.

To predict the cis-acting elements among the *PtEPF/EPFL*, the 2,000 bp upstream promoter sequence of EFP genes was collected and analyzed using PlantCARE. As shown in Figure 6, the cis-acting elements associated with hormonal response, stress response elements, light response, growth and development-related elements, site binding and other functional categories. The hormonal response elements include salicylic acid response elements (SARE and TCA-element), the ABA response element (ABRE), the auxin response elements (AuxRR-core and TGA-element), the P-box and the gibberellin response elements (GARE-motif), CGTCA-motif and TGACG-motif related to jasmonic acid (MeJA). Additionally, the GC-motif, low temperature response element (LTR), the defense and stress response element (TC-rich repeats), the anaerobic induction element (ARE) and the MYB binding site involved in light responsiveness (MBS) were associated with stress responsiveness. Growth and development-related elements comprise the GCN4_motif related to endosperm expression, the CAT-box associated with meristem expression, and the zein metabolism regulation (O2-site). Light responsive elements include G-box, 3-AF1 binding site and GT1-motif.

### 3.7 Analysis of gene expression characteristics of *PtEPF/EPFL* family genes

**FIGURE 7:**
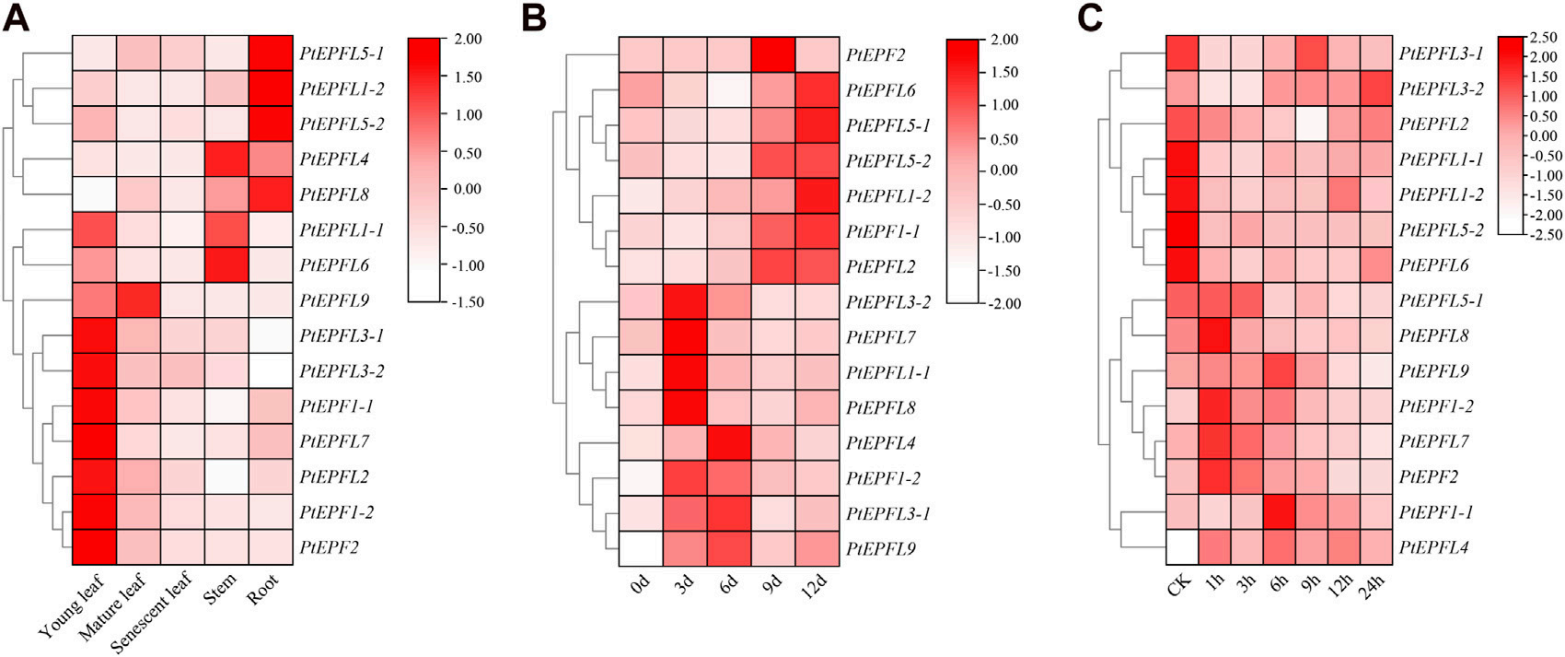
The expression pattern of *PtEPF/EPFL*. **(A)** Heatmap showing the tissue-specific expression of *PtEPF/EPFL* genes. “Young leaf”, the first one to three leaves from the shoot apex; ‘‘mature leaf”, a fully expanded leaf; “senescent leaf”, basal most two to three leaves above the root system. **(B)** Expression pattern of *PtEPF/EPFL* genes under drought stress. **(C)** Expression pattern of *PtEPF/EPFL* genes under ABA treatment. Data represent mean ± SE (n = 3).

To explore the potential biological roles of *PtEPF/EPFL*, qRT-PCR was employed to assess the relative transcription levels of *PtEPF/EPFL* in different tissues of poplar. The result showed that the expression of *PtEPF/EPFL* has obvious tissue specificity (Figure 7A). Among them, *PtEPF1-1*, *PtEPF1-2*, *PtEPF2*, *PtEPF3-1*, *PtEPF3-2*, *PtEPFL6* and *PtEPFL7* were mainly expressed in young leaves, *PtEPFL1-1* was mainly expressed in young leaves and stems, *PtEPFL1-2*, *PtEPFL5-1*, *PtEPFL5-2* and *PtEPFL8* were mainly expressed in roots, *PtEPFL4* and *PtEPFL6* was mainly expressed in stems, and *PtEPFL9* was highly expressed in young and mature leaves.

In order to identify the response of *PtEPF/EPFL* family genes to stress treatment, poplar plants were subjected to drought and ABA stress treatment. Then the expression of these 15 genes was quantitatively analyzed. The expression pattern of *PtEPF/EPFL* gene was different between drought stress and ABA treatment. As can be seen from Figure 7B, the expression of *PtEPF/EPFL* family genes were basically increased after drought stress treatment, but the space and time of the increase were different. The expression levels of *PtEPF1-2*, *PtEPFL1-1*, *PtEPFL3-2*, *PtEPFL7* and *PtEPFL8* reached their peak at 3 days of drought stress, and the expression of *PtEPF1-2* was the highest. The expression levels of *PtEPFL3-1*, *PtEPFL4* and *PtEPFL9* were highest at 6 days after drought stress. The expression levels of *PtEPF2* and *PtEPFL2* were highest at 9 days after drought stress. *PtEPF1-1*, *PtEPFL1-2*, *PtEPFL5-1*, *PtEPFL5-2*, and *PtEPFL6* expressed highest at 12 days after drought stress. ABA is an important stress hormone in plants, which can regulate plant stress adaptation to drought and other environmental factors. As can be seen from Figure 7C, some *PtEPF/EPFL* family genes were upregulated and some were downregulated after ABA spraying treatment. The expression levels of *PtEPF1-1*, *PtEPF1-2*, *PtEPF2*, *PtEPFL7* and *PtEPFL8* were rapidly upregulated after spraying ABA, while the expression levels of *PtEPFL1-1*, *PtEPFL1-2*, *PtEPFL5-2* and *PtEPFL6* were significantly downregulated. The expression levels of other genes were not significantly responsive to ABA.

## 4 Discussion

As an important channel for gas exchange (CO_2_ and water vapor) between the external environment and plants, stomata play a crucial role in regulating plant photosynthesis and transpiration (Bertolino et al., 2019; Buckley et al., 2019). As a kind of epidermal model factor, EPF gene family can encode a specific secretory polypeptide in plants and play an important role in stomatal development (Takata et al., 2013; Uchida and Tasaka, 2013). Through identification and analysis of poplar EPF gene family, the characteristics and functions of poplar EPF gene were revealed.

In this study, 15 *EPF* genes were identified in poplar and the *PtEPF/EPFL* family members are characterized by relatively short protein lengths and similar molecular weights (Table 1). According to the phylogenetic tree shown in Figure 3, these results support the classification of 15 PtEPF members into four subfamilies (I, II, III, and IV). The analysis of the phylogenetic tree of plants of different evolutionary levels showed that the stomata and vascular tissues had not evolved in the lower bryophytes and ferns, but members of the EPF family had appeared. EPF1-EPF2-EPFL7 and EPFL4-6 branches contain genes from bryophytes and genes from vascular plants, while EPFL1-3-EPFL8 and EPFL9/Stomagen branches contain no genes from bryophytes and are composed of genes from vascular plants. EPF family members have increased during evolution possibly due to the duplication of genes or genomes. EPFs have six conserved cysteine residues in the C-terminal region of the mature peptide, in addition to two additional conserved cysteine residues in the EPF1-EPF2-EPFL7 branch (Figure 4). Disulfide bonds can be formed between cysteines. Shimada et al. speculated that this may be the site where the proteins of this family interact with downstream protein kinase receptors (Shimada et al., 2011). The promoter region of the *PtEPF/EPFL* family gene has been analyzed predictively, revealing the presence of various elements responsible for hormonal response, stress response, growth and development, light response, site binding, and other functional categories. This suggests that the *PtEPF/EPFL* gene family exhibits functional diversity due to the presence of these diverse regulatory elements.

The results of tissue expression revealed that most *PtEPF* genes exhibited expression across all organs (Figure 7A). Specifically, *PtEPF1-1*, *PtEPF1-2*, *PtEPF2*, and *PtEPFL9*, which are homologous to the *AtEFPL9*, *AtEFP1* and *AtEFP2* genes that regulate stomatal development in *Arabidopsis* (Hara et al., 2007; Hara et al., 2009; Hunt et al., 2010), showed high expression levels in leaves. The expression levels of *PtEPFL4*, *PtEPFL5-1*, *PtEPFL5-2*, and *PtEPFL6* were notably elevated in both roots and stems. This observation aligns with the known function of the CHAL subfamily, which primarily revolves around promoting plant growth (Abrash et al., 2011; Uchida et al., 2012). The expression of *EPF* family genes were basically increased after drought stress treatment (Figure 7B). It is speculated that these genes play an important role in drought resistance. Drought stress induced the accumulation of ABA in plant cells, in response to the expression of ABA regulatory genes, resulting in stomatal closure, thereby improving the drought resistance of plants (Wang et al., 2021). The signal pathways of plants responding to drought stress can be divided into ABA-dependent signaling pathways and ABA-independent signaling pathways (Park et al., 2016). Further analysis of *PtEPF* genes response to ABA stress showed that the *PtEPF1-1*, *PtEPF1-2*, *PtEPF2*, *PtEPFL7* and *PtEPFL8* genes were induced to increase expression after ABA spraying treatment, while the *PtEPFL1-1*, *PtEPFL1-2*, *PtEPFL5-2* and *PtEPFL6* were downregulated of gene expression (Figure 7C). The expression patterns of the genes were different under drought or ABA treatment, suggesting that these *PtEPF/EPFL* genes may have regulatory functions in the tolerance to drought and ABA stress and deserve further investigation.

## 5 Conclusion

In populus genome, we identified 15 EPF genes and analyzed the gene structure, genome location, phylogenetic relationship, conserved domain of proteins, promoter elements and gene expression using bioinformatics methods. Our results provided important information on the functional role of EPF genes in populus. Generally, studying the molecular characteristics of *PtEPF/EPFL* contributes to the understanding of their specific biological functions, which provided a theoretical basis for breeding poplar varieties with high water use efficiency and fast growth rate.

## Data Availability

The original contributions presented in the study are included in the article/Supplementary Material, further inquiries can be directed to the corresponding author.
