# Supplementary material for: Genome-wide identification and expression analysis of EPF/EPFL gene family in Populus trichocarpa
Source: Front Genet. 2024 Jul 18;15:1432376. doi: 10.3389/fgene.2024.1432376 (PMC11291230; doi:10.3389/fgene.2024.1432376)
Supplement: Supplementary file 1 [file Table1.DOCX]

**Supplementary Table 1 Gene-specific primers used for qRT-PCR assays**

| Gene name | Primer Sequence (5’to 3’) |
| --- | --- |
| *PtEPF1-1* | Forward: 5’-ATGCTCACCATGTAGACTAG-3’  Reverse: 5’-GGACAGGATAAGACTTGTTATG-3’ |
| *PtEPF1-2* | Forward: 5’-TCTCTGTCTTCTTCCAATGG-3’  Reverse: 5’-CAAGTCTCAGCCTCTTCAA-3’ |
| *PtEPF2* | Forward: 5’-CAAGGAAGCAGCAAGAGA-3’  Reverse: 5’-TGAAGGCACATGGTAGTATT-3’ |
| *PtEPFL1-1* | Forward: 5’-AGGAGAAGGCAAGGCTAG-3’  Reverse: 5’-TGATTCGGCAGAGTAGGC-3’ |
| *PtEPFL1-2* | Forward: 5’-TCTTGTCGCCTGTTACCT-3’  Reverse: 5’-TAGCCTTGCCTTCTCCTC-3’ |
| *PtEPFL2* | Forward: 5’-TTGGCTTCAGTTGTCACA-3’  Reverse: 5’-ACTTCTCTTACGGCTATGG-3’ |
| *PtEPFL3-1* | Forward: 5’-TCCACTGGACTCGAACTT-3’  Reverse: 5’-GATGGCTGGCACTTGAAT-3’ |
| *PtEPFL3-2* | Forward: 5’-GGACACAGCCTTCAATCTT-3’  Reverse: 5’-CTGCTGCCTCAATTATCATC-3’ |
| *PtEPFL4* | Forward: 5’-TTCAGCACCATCTCTTCC-3’  Reverse: 5’-GTAATACTCAGCAGTCACC-3’ |
| *PtEPFL5-1* | Forward: 5’-CACCGTCACCACTTCTTAT-3’  Reverse: 5’-CTCGTTGCCACACTTACA-3’ |
| *PtEPFL5-2* | Forward: 5’-ACCACTTATCAACACTCACT-3’  Reverse: 5’-TTACATCTCCAAGCCTCTG-3’ |
| *PtEPFL6* | Forward: 5’-GTCTCCTTCACCATCTTCTT-3’  Reverse: 5’-CATCATAACCTCTTGTCTTCTC-3’ |
| *PtEPFL7* | Forward: 5’-GGAGGCTGACACTAACTTC-3’  Reverse: 5’-CACAAGCATGAGAGCAATC-3’ |
| *PtEPFL8* | Forward: 5’-GCGACAGACTCCTCATAC-3’  Reverse: 5’-AGAGCACCTATTGACACATT-3’ |
| *PtEPFL9* | Forward: 5’-TTCACCACCTGCTCATAAG-3’  Reverse: 5’-GACATCTGCTCTTCATTGC-3’ |
| *PtUBQ* | Forward: 5’-AGACCTACACCAAGCCCAAGAAGAT-3’  Reverse: 5’-CCAGCACCGCACTCAGCATTAG-3’ |
